# Research Progress on the Effects of Support and Support Modification on the FTO Reaction Performance of Fe-Based Catalysts

**DOI:** 10.3390/molecules28237749

**Published:** 2023-11-24

**Authors:** Yuan Wen, Chenliang Zhou, Linfei Yu, Qiang Zhang, Wenxiu He, Quansheng Liu

**Affiliations:** 1 Inner Mongolia Key Laboratory of Coal Chemical Industry and Comprehensive Utilization, Inner Mongolia University of Science & Technology, Baotou 014010, China; 15847250829@163.com (Y.W.); ff15319423704@163.com (L.Y.); nmghwx@sina.com (W.H.); 2 Baotou Huan Run Environmental Protection Investment Co., Ltd., Baotou 014010, China; vip-no1-80291125@163.com; 3 Inner Mongolia Key Laboratory of High-Value Function Utilization of Low Rank Carbon Resource, Inner Mongolia University of Technology, Hohhot 010051, China; liuqs@imut.edu.cn

**Keywords:** Fischer–Tropsch to olefins, iron-based catalyst, metallic oxide, molecular sieve, carbon material, hydrophobic modification

## Abstract

In recent years, the non-petroleum production of light olefins has been the research focus of Fischer–Tropsch olefin synthesis (FTO). Iron-based catalysts have attracted much attention because of their low price, high catalytic activity, and wide temperature range. In this paper, traditional modification, hydrophobic modification, and amphiphobic modification of the catalyst are summarized and analyzed. It was found that traditional modification (changing the pore size and surface pH of the catalyst) will reduce the dispersion of Fe, change the active center of the catalyst, and improve the selectivity of light olefins (for example, SiO_2_: 32%). However, compared with functional methods, these traditional methods lead to poor stability and high carbon dioxide selectivity (for example, SiO_2_: 34%). Hydrophobic modification can inhibit the adsorption and retention of water molecules on the catalyst and reduce the local water pressure near the iron species in the nuclear layer, thus inhibiting the further formation of CO_2_ (for example, SiO_2_: 5%) of the WGSR. Amphiphobic modification can not only inhibit the WGSR, but also reduce the steric hindrance of the catalyst, increase the diffusion rate of olefins, and inhibit the reabsorption of olefins. Follow-up research should focus on these issues.

## 1. Introduction

Fossil energy is the main resource in the world today. The current world energy structure is dominated by oil, coal, and natural gas. However, with increasing international oil prices, the development and utilization of other energy is a top priority. As the world’s second largest energy source, the use of coal is gradually increasing. The traditional way to use coal is to burn it, which has caused a great negative impact on the environment. With the continuous development of the social economy and the adjustment of energy structure, using coal efficiently and greenly has become one of the urgent problems to be solved in the world [1,2].

Light olefins (C_2_^=^~C_4_^=^) are the key raw material of the basic chemical industry. Its output measures the development level of a country’s chemical industry and also drives the development of other organic industries. With the development of the economy, people’s demand for light olefins is increasing [3,4]. Therefore, it is necessary to develop a technology to produce light olefins from non-petroleum resources such as coal, natural gas, or biomass to replace the traditional catalytic cracking naphtha [5]. At present, non-petroleum routes to produce light olefins mainly include Oxidative Coupling of Methane (OCM), CO_2_ hydrogenation, the Fischer–Tropsch to olefins (FTO) reaction, and so on [6]. Among these pathways, the FTO reaction has the most technical, economical, safe, and energy-saving characteristics [3]. Using syngas as a raw material to produce light olefins can be divided into direct and indirect methods. Among them, the indirect method has been industrialized in many areas. The reaction equation is as follows: CO + H_2_ → 2CH_3_OH ↔ CH_3_OCH_3_ → C_2_^=^~C_4_^=^. However, the indirect method has some disadvantages, such as complex steps, long technological processes, high energy consumption, and so on [5]. Therefore, in order to overcome the shortcomings of the indirect method, the direct method (namely the FTO reaction) to produce light olefins has become a research hotspot. However, in order to commercialize the indirect method as soon as possible, it is necessary to develop a catalyst with a low price and a good reaction performance. Fe-based catalysts have attracted much attention because of their low cost, high catalytic activity, wide temperature range, and high olefin selectivity. The kinetics of parallel reactions, such as the hydrogenation of CO, the water gas shift reaction (WGSR: CO + H_2_O = CO_2_ + H_2_), and the secondary reaction of olefins, determine the overall selectivity of the FTO product [6].

**Figure 1:**
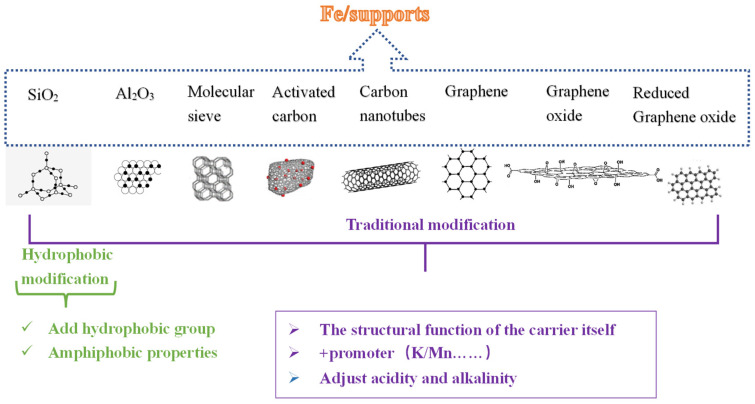
Supports of iron-based catalysts in the FTO reaction.

Gao et al. [7] found that more and more studies have focused on the addition of promoters and the modification of supports in recent years. The introduction of promoters and the optimization of supports will achieve moderate hydrogenation, control C–C coupling, improve the selection of the catalyst target products (C_2_^=^~C_4_^=^), and achieve the stability and durability of the reaction. At the same time, it was also found that olefins are prone to secondary hydrogenation at the active site, which brings a variety of adverse effects to the FTO reaction, such as low CO conversion, low content of light olefins in the product, and serious carbon deposition in the Fe-based catalyst. The selectivity of CO_2_ byproducts in the FTO process is usually in the range of 30% to 50%, which significantly reduces the carbon utilization efficiency [8,9,10]. CO_2_ is mainly produced by the WGSR. Considering the idea of limiting the WGSR, more and more researchers have begun to use methyl groups to modify the surface of the support to form a hydrophobic shell in recent years [11,12,13], which will greatly reduce CO_2_ selectivity. Although the secondary hydrogenation ability of Fe-based catalysts is relatively weak, some oily compounds (C_6_~C_15_ organic compounds) are still generated during the reaction process [12]. Therefore, in order to further improve the effective utilization rate of C on the basis of reducing CO_2_ selectivity, some researchers have tried to coat the support with hydrophobic and oleophobic materials to achieve the purpose of reducing CO_2_ selectivity and reducing the selectivity of oil-like compounds. Among them, fluorine-containing compounds with low surface energy are used to prepare amphiphobic surfaces due to their excellent chemical stability, high surface activity, hydrophobicity, and oleophobicity [14]. Therefore, as shown in Figure 1, the effects of traditional modification, hydrophobic modification, and amphiphobic modification on metal oxides, molecular sieves, and carbon materials are discussed, and the effects of the promoters, support structure, and support modification on the reaction performance of the catalyst are analyzed.

In this paper, for the first time, the authors compared the results of previous studies at the same computational baseline level, taking into account CO_2_ selectivity. Therefore, the problem of incomparable results caused by inconsistent calculation benchmarks is avoided. The influence of hydrophobic modification on product distribution and its mechanisms are explored.

## 2. Calculation Formulas

In the existing literature reports, the calculation of reaction performance mainly includes CO conversion, CO_2_ selectivity, hydrocarbon product distributions, and hydrocarbon selectivity. The calculation formulas of CO conversion and CO_2_ selectivity are consistent in all the literature reports, as shown in Equations (1) and (2), respectively. However, different formulas were used to calculate the distributions and/or selectivity of the hydrocarbon products. For example, Equations (3) or (4) are expressed as the mass fraction or C atom fraction of a particular hydrocarbon in the total hydrocarbons, respectively. Strictly speaking, the results obtained by Equation (3) or Equation (4) should be called hydrocarbon product distributions, and should not be written as hydrocarbon selectivity. Except for the problems mentioned above, there is another reason that the catalytic performance is not comparable or referable, namely, the quality of the active metal components is not consistent. In recent years, lots of scholars have paid attention to the aforementioned problems and calculated the reaction performance based on a 1 g Fe atom using Equations (5) and (6). The detailed calculation process is shown in our previous literature [15].

(1) $$X_{\mathrm{CO}}=\frac{M_{\mathrm{in}}^{\mathrm{CO}}-M_{\mathrm{out}}^{\mathrm{CO}}}{M_{\mathrm{in}}^{\mathrm{co}}}\times 100\%$$

(2) $$S_{{\mathrm{CO}}_{2}}=\frac{M_{\mathrm{out}}^{{\mathrm{CO}}_{2}}}{M_{\mathrm{in}}^{\mathrm{CO}}-M_{\mathrm{out}}^{\mathrm{CO}}}\times 100\%$$

(3) $$S_{C_{n},m,d}=\frac{m_{C_{n}}}{\sum_{1}^{n}m_{C_{n}}}\times 100\%,\left(n=1,2,3,4\right)$$

(4) $$S_{C_{n},C,d}=\frac{n_{i}M_{\mathrm{out}}^{i}}{\sum_{1}^{n}n_{i}M_{\mathrm{out}}^{i}}\times 100\%,\left(n=1,2,3,4\right)$$

(5) $$S_{C_{n}}=\frac{n_{i}M_{\mathrm{out}}^{i}}{M_{\mathrm{in}}^{\mathrm{CO}}-M_{\mathrm{out}}^{\mathrm{CO}}}\times 100\%,\;(n=1,2,3,4)$$

(6) $$S_{C_{5}{}^{+}}=100-\sum S_{\mathrm{Cn}},\;(n=1,2,3,4)$$

In order to compare the reaction performance of catalysts reported in different literatures, the data under different benchmarks are uniformly transformed into basic 1 g Fe atom and C atom fractions using Equations (7) and (8).

The product distribution expressed by the C atom fraction was transformed into hydrocarbon selectivity using Equation (7):

(7) $$S_{\mathrm{Cn}}=\frac{n_{i}M_{\mathrm{out}}^{i}}{M_{\mathrm{in}}^{\mathrm{CO}}-M_{\mathrm{out}}^{\mathrm{CO}}}\times 100\%=\left(\frac{100-S_{{\mathrm{CO}}_{2}}}{100}\right)\times S_{C_{n},C,d}\times 100\%,\;(n=1,2,3,4)$$

The product distribution expressed by the mass fraction is transformed into hydrocarbon selectivity using Equation (8):

(8) $$S_{\mathrm{Cn}}=\frac{n_{i}M_{\mathrm{out}}^{i}}{M_{\mathrm{in}}^{\mathrm{CO}}-M_{\mathrm{out}}^{\mathrm{CO}}}\times 100\%=\left(\frac{m_{\mathrm{Cn}}/W_{\mathrm{Cn}}}{\sum_{1}^{n}m_{\mathrm{Cn}}/W_{\mathrm{Cn}}}\right)\left(\frac{100-S_{{\mathrm{CO}}_{2}}}{100}\right)\times 100\%,\;(n=1,2,3,4)$$

where $M_{\mathrm{in}}^{\mathrm{CO}}$ is the inlet carbon number of CO, $M_{\mathrm{out}}^{\mathrm{CO}}$ and $M_{\mathrm{out}}^{{\mathrm{CO}}_{2}}$ are the carbon numbers of CO and CO_2_ in the tail gas, $W_{\mathrm{Cn}}$ is the molecular mass of a hydrocarbon with n carbon atoms, i is for a particular hydrocarbon, and n (n = 1, 2, 3, 4) is the carbon number of the hydrocarbon.

## 3. Metallic Oxide

### 3.1. SiO_2_

SiO_2_ mainly affects the dispersion of active components, adsorption and desorption of reactants and products, reduction, carbonization, mechanical strength, and textural properties of catalysts. In addition, a strong metal–support interaction (SMSI) can also change the reaction performance of the catalyst by affecting the acidity and alkalinity of the catalyst surface [7].

(1) SMSI

**Figure 2:**
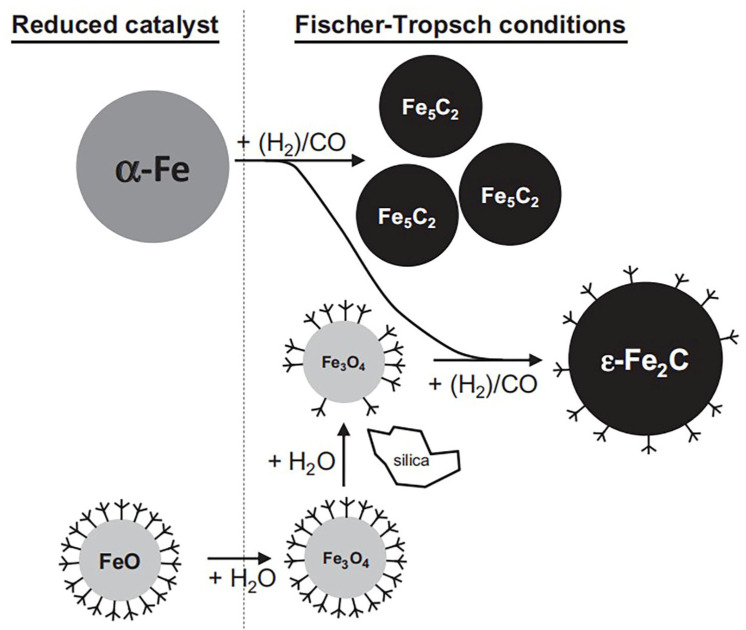
Transformation of *α*-Fe and FeO stabilized by silicate surface ligands during the Fischer–Tropsch synthesis [18].

SiO_2_ has strong acid resistance, heat resistance, wear resistance, low surface acidity, and a porous structure, and is widely used as the support of the FTO catalyst. There is a strong interaction between SiO_2_ and metallic Fe, which increases the difficulty of O removal, reduces the reduction and carbonization of the catalyst, improves the WGSR, and inhibits the reaction performance of the catalyst [16,17]. Mogorosi et al. [18] used surface silicate groups to modify the surface of nanometer iron oxide crystal and studied the metal–support interaction between Fe and SiO_2_ in an Fe-based catalyst. It is difficult to remove O^2−^ from syngas and eliminate the byproduct of water without modifying the catalyst surface [19,20]. And water can form CO_2_ through the WGSR again, which enhances the WGSR and makes the CO_2_ selectivity higher. After, the ligands introduced by the silicon modification lower the surface energy of the nano-sized FeO thus disallowing the disproportionation into magnetite and *α*-Fe thermodynamically. Thus, the WGSR is suppressed. The presence of silicate groups on the surface will also weaken the adsorption of CO, which leads to a higher hydrogen utilization rate on the catalyst surface and in turn, leads to increased CO conversion, an increase in methane selectivity, and a decrease in light olefin selectivity, as shown in Figure 2. Suo et al. [16] studied the effect of SiO_2_ on the chemical properties and reaction performance of Fe-based catalysts by preparing Fe/SiO_2_ catalysts with different molar ratios, as shown in Table 1 (Fe_1_Si, Fe_10_Si, and Fe_25_Si). The CO conversion first decreased, passed through a minimum at Fe_10_Si (34%), and then sharply increased to a high level (53%). The decreased activity with increasing silica content led to a lower degree of reduction and a reduction in the effective potassium content of the catalyst. It was found that with reduction or activation, a small amount of silica (Fe_25_Si) inhibits the reduction or carburization of catalysts, while a large amount of silica (Fe_1_Si) improves the reduction or carburization of iron oxides. The interaction between SiO_2_ and Fe will form the Fe_2_SiO_4_ phase during the FTO reaction, which further changes the electronic structure of Fe atoms in FeO and inhibits the reduction and activation of the catalyst. The main reason for inhibiting reduction might be the increasing WGSR and the continuous formation of water in the catalyst, which causes the iron carbide to be continuously oxidized [21,22]. Therefore, the selectivity of CO_2_ is the lowest (12%).

(2) The structural function support

The pore size and porosity of the support have a certain relationship with the performance of the catalyst. Cheng et al. [23] studied the effect of the support’s pore size on the performance of the catalyst and found that the carbonation reaction depends on the pore size of SiO_2_, as shown in Table 1 (10FeSi17.5). Fe_2_O_3_ distributed on the surface of SiO_2_ with a large pore size more easily reacts with CO (the kinetic diameters of CO, CO_2,_ and H_2_ are 0.38, 0.33, and 0.29 nm, respectively) [24,25], which can preferentially generate the active species of Fe_x_C (*θ*-Fe_3_C, *χ*-Fe_2.5_C, *ε*′-Fe_2.2_C, *ε*-Fe_2_C) for chain growth, CO decomposition, and hydrogenation [26], and improve the reactivity and selectivity of light olefins. On the contrary, Fe_2_O_3_ loaded on a SiO_2_ surface with a small pore size is not conducive to the formation of Fe_x_C, thus does not contribute to improving the reaction performance. When the pore size of SiO_2_ reaches 17.5 nm, the selectivity of light olefins is the highest (15%), CO conversion is 28%, and the yield of light olefins is 4%. Liu et al. [27] studied the influence of the pore size of the supports and the particle size of Fe_2_O_3_ (by X-ray diffraction before synthesizing the catalyst) on the reaction performance of the catalysts, as shown in Table 1 (FeMnS5, FeMnS50, FeMnS80, and FeMnS80E). With an increase in pore size (5, 50, and 80 nm), the selectivity of light olefins is 23%, 29%, and 32%, respectively. Meanwhile, the CO conversion (*X*_CO_ = 44%) reaches its peak when the pore size is 50 nm. The fundamental reason for this is that the increase in diffusion efficiency in a larger pore size inhibited the secondary reaction of light olefins, resulting in an increase in light olefin selectivity with the increase in pore size [28,29,30,31,32,33,34]. The particle size of Fe_2_O_3_ has a significant effect on the CO conversion and the selectivity of light olefins. This indicated that the smaller Fe_2_O_3_ particle sizes favors the generation of more active sites of Fe_x_C. Nevertheless, neither the pore size nor the particle size has an obvious effect on the CO_2_ selectivity when the pore size is larger than 50 nm. Suo et al. [16] found that the addition of SiO_2_ increases the dispersion of Fe, changes the active sites of the catalyst, increases the specific surface area of the catalyst, and increases the anti-sintering and wear resistance of the catalyst. However, it was also found that with the increase in Si content, Fe_x_C is inhibited at first and then promoted. To a large extent, water can promote the oxidation of Fe_x_C to FeO and C, thus contributing to the formation of carbon deposition, reducing the CO conversion of the catalyst and the selectivity of light olefins. However, the formation of the FeO layer on the catalyst surface can control the water concentration on the catalyst surface, inhibit the WGSR, stabilize the iron grain, and prevent its oxidation [35,36]. In addition, the influence of catalysts on the reaction performance and the WGSR can also be studied by preparing different catalyst structures. Zhang et al. [37] studied the structure–property relationship between Fe and SiO_2_ by preparing a nested Fe@Si catalyst, an impregnated Fe/Si catalyst, and a precipitated Fe-Si catalyst, as shown in Table 1 (Fe@Si, Fe/Si, and Fe-Si). Compared with Fe@Si catalysts, Fe/Si and Fe-Si catalysts effectively improve the confinement effect of the SiO_2_ shell, so the water can be removed quickly. However, the Fe@Si catalyst has the strongest anti-sintering performance. Due to the limitation of the SiO_2_ shell, water is produced and local water pressure is increased, which strengthens the WGSR and hinders the reduction process of iron oxide. The deep oxidation of Fe_x_C deactivated the Fe@Si catalyst. Therefore, the CO conversion of the Fe@Si catalyst decreased from 67% to 48% after 64 h of the reaction. For the catalysts obtained from the traditional method (Fe/Si and Fe-Si), the reduction process of iron oxide was improved since the H_2_O removal efficiency was high. Compared with Fe@Si and Fe/Si, the Fe-Si catalyst owned the lowest CO_2_ selectivity, CO conversion, and light olefins selectivity of 11%, 30%, and 13%, respectively, due to the strongest Fe–SiO_2_ interaction in the Fe-Si catalyst. Meanwhile, among the three catalysts, the Fe/Si catalyst had the highest selectivity for light olefins (19%).

(3) Surface acidity and alkalinity of the supports

SiO_2_ mainly exists in an amorphous state in the catalyst and has a certain acidity, which can disperse and stabilize the iron phase [16]. At the same time, changing the surface acidity and alkalinity of the support can also affect the reaction performance of the catalyst [38,39]. Wan et al. [38] compared the catalytic performance of Fe/Cu/K catalysts with and without SiO_2_ under fixed bed reaction conditions (533 K, 1.5 Mpa, 1000 h^−1^, and H_2_/CO = 2). It was found that the surface alkalinity and initial activity of the catalyst decreased after adding SiO_2_. Das et al. [39] prepared Fe-Cu/SiO_2_ catalysts with different K contents using co-precipitation and wet impregnation, as shown in Table 1 (Cat._0_K, Cat._0.5_K, Cat._1_K, Cat._3_K, Cat._5_K). It was found that the addition of the alkali metal promoter K can increase alkaline sites in the catalyst, reduce the acidity of the support, and then weaken the strength of the C-O bond, reinforcing the dissociation and hydrogenation capacity of CO. As a result, the light olefin selectivity of the catalysts with the K addition (Cat._0.5_K, Cat._1_K, Cat._3_K, Cat._5_K) is significantly higher than that of the same without a K addition (Cat._0_K). CO conversion (56%) and light olefin selectivity (about 13%) reach their peak together when the K content is 1 wt%. The addition of K can increase the surface alkaline site of the catalyst, reduce the acid site, and then enhance the adsorption and dissociation for CO, hindering the adsorption and dissociation for H_2_. With increased potassium loading of more than 1 wt% on the catalyst, the CO dissociative and associative adsorption is significantly increased, leading to the deposition of carbon on the surface, and the H_2_ chemisorption is suppressed, which results in a decline in CO-hydrogenation activity for Cat._3_K and Cat._5_K.

The reaction performance of the catalyst is related to the following functional structure of the catalyst. Firstly, when the pore size of the support is larger than a certain characteristic size, the reaction performance of the catalyst will not be affected due to the disappearance of steric hindrance and the domain limiting effect. Secondly, the smaller the particle size of the active component, the better the increase in the number of defect sites, particle edges and angles, specific surface area, and active center exposed on the surface of the active component. Thirdly, the structure of the catalyst also plays a good role in regulating the surface properties of the active components (including electron migration, SMSI, synergistic effect, and so on). Finally, appropriately increasing the basic sites on the catalyst surface can promote the adsorption and dissociation of CO and reduce the H_2_/CO molar ratio on the catalyst surface.

**Table 1:** Summary of FTO performance of iron catalysts supported on SiO_2_.

Catalysts	Temperature (K)	Pressure (MPa)	H_2_/CO	$X_{\mathrm{CO}}$ (%)	$S_{C_{n}}$ (%C mol)	$S_{{\mathrm{CO}}_{2}}$ (%)	Yield (%)	Reference	Eqs.
Fe_1_Si	553	1.5	2	45	8	21	4	[16]	(8) ^a,b^
Fe_10_Si	553	1.5	2	34	8	12	3	[16]	(8) ^a,b^
Fe_25_Si	553	1.5	2	53	10	18	5	[16]	(8) ^a,b^
10FeSi17.5	573	2.0	2	29	15	11	4	[23]	(4) ^b^
FeMnS5	573	1.0	1	22	23	24	5	[26]	(7) ^b^
FeMnS50	573	1.0	1	44	29	34	13	[27]	(7) ^b^
FeMnS80	573	1.0	1	40	32	34	13	[27]	(7) ^b^
FeMnS80E	573	1.0	1	51	36	33	18	[27]	(7) ^b^
Fe@Si	553	2.0	2	48	18	21	9	[37]	(4) ^b^
Fe/Si	553	2.0	2	56	19	22	11	[37]	(4) ^b^
Fe-Si	553	2.0	2	30	13	11	4	[37]	(4) ^b^
Cat_0_K	518	1.5	2	38	6	12	2	[39]	(4) ^c^
Cat_0.5_K	518	1.5	2	45	10	13	4	[39]	(4) ^c^
Cat_1_K	518	1.5	2	5	13	18	7	[39]	(4) ^c^
Cat_3_K	518	1.5	2	39	12	20	5	[39]	(4) ^c^
Cat_5_K	518	1.5	2	28	13	22	4	[39]	(4) ^c^

^a^: In the calculation of Equation (8), C_3_H_6_ and C_5_H_10_ are used to represent C_2_~C_4_ and C_5_^+^, ^b^: Results are not based on 1 g Fe, ^c^: Results are based on 1 g Fe.

### 3.2. Al_2_O_3_

Al_2_O_3_ is a common metal oxide support for the FTO reaction. It has the characteristics of a high melting point, strong thermal stability, large specific surface area, and low cost [40].

(1) SMSI

There is also a strong interaction between Al_2_O_3_ and Fe, which affects the reaction performance of the catalysts. Wan et al. [41] researched the influence of the interaction between Fe and Al_2_O_3_ on the reaction performance of the Cu-K-Fe/Al_2_O_3_ catalyst. It indicated that the strong interaction between Fe and Al_2_O_3_ can reduce the surface alkalinity of the catalyst, inhibit the adsorption of CO, and reduce the reduction and carburization and activity of the catalyst in the CO + H_2_ atmosphere. SMSI also inhibits the WGSR, reduces CO_2_ selectivity (as shown in Table 2, Fe and Fe/Al_2_O_3_), stabilizing the active sites of the Fe-based catalyst. On the contrary, Wang et al. [42] illustrated that with an increase in MgO content, the strength of the medium basic sites (caused by Mg^2+^/O^2−^) gradually deteriorated due to the formation of MgAlO_4_ spinel, and the surfaces of the support and catalyst were mainly composed of weakened medium basic sites and weak basic sites (caused by surface hydroxyls). The formation of MgAlO_4_ spinel weakens the interaction between the active iron and the support, promotes the conversion of iron species to active iron, and then promotes the formation of light hydrocarbons [43]. Meanwhile, the weakened medium and weak basic sites promote the desorption of olefin species, which is beneficial for increasing the selectivity of light olefins in the products. However, when the mole ratio of MgO is more than 50%, the enhanced basicity promotes the interaction between the active iron and the support, which inhibits hydrocarbon desorption, leading to the growth of carbon chains. It is worth noting that the selectivity of CO_2_ changed a little from 49% (Al_2_O_3_) to 46% (50%MgO/Al_2_O_3_), and was almost constantly 46% when there was over 50% to 100% of MgO content, as shown in Table 2 (Al_2_O_3_, 50%MgO/Al_2_O_3_, MgO).

(2) Structural function

Wan et al. [44] found that when Al_2_O_3_ was added to the Fe-Cu-K catalyst using the precipitation method (CA-0:100Fe/6Cu/5K, CA-2:100Fe/6Cu/5K/15Al_2_O_3_), a high dispersion of Fe_2_O_3_ crystals could be promoted, the specific surface area could be further increased, the pore diameter could be reduced (CA-0:33.8 nm → CA-2:24.2 nm), the adsorption capacity of H_2_ could be improved, and the WGSR could be reduced (extent of WGSR, CA-0:116.3 → CA-2:0.4). Numpilai et al. [28] studied the effect of the pore size of an Al_2_O_3_ support on the reaction performance of Fe-based catalysts, as shown in Table 2, where S-Al_2_O_3_, M-Al_2_O_3_, and L-Al_2_O_3_ represent pore sizes of 6.2 nm, 49.7 nm, and 152.3 nm, respectively. With an increase in pore size, the selectivity of the light olefins is 38, 41, and 41%. The selectivity of the light olefins reaches the minimum (38%) on the S-Al_2_O_3_ catalyst. The main reason for this is that the small pore size of the catalyst limits the diffusion of H_2_, making it impossible to fully react in the small pore size to form light olefins.

(3) Synergetic effect

Galis et al. [45] added a small amount of the Na + S promoter (about 0.2 wt% Na and 0.03wt% S) into the Fe-Al_2_O_3_ catalyst to study the influence of the promoter on the reaction performance of the catalyst, as shown in Table 2 (5AFe and 5AFe-S-Na). It was found that under the conditions of low pressure (1 bar), the selectivity of the light olefins increased from 19% (5AFe) to 28% (5AFe-S-Na). The main reason for this is that Na can inhibit the secondary hydrogenation of olefins, increase the chain growth probability, and reduce methanation by adjusting the electronic structure of the active substances. S can selectively mask part of the hydrogenation active sites, weaken the Fe–CO bond on the surface of the catalyst, and improve the CO conversion of the catalyst and the selectivity of light olefins [46,47]. However, Liu et al. [48] added Al_2_O_3_:ZnAl_8_O_13_ in the ratio of 3:1 to the Fe-K catalyst, as shown in Table 2 (Fe-K_2_O/ZnAl_8_O_13_). It was found that under high pressure (2.0 MPa), the selectivity of light olefins is 30%. Simultaneously, its contents of ethylene in C_2_ (i.e., the C_2_ olefin fraction), propylene in C_3_ (i.e., the C_3_ olefin fraction), and butylene in C_4_ hydrocarbons (i.e., the C_4_ olefin fraction) achieve 88, 92, and 89%, respectively. The selectivity of C_5_^+^ is 6%. The main reason for this is that the synergistic effect of high-temperature passivated ZnAl_2_O_4_ and alumina inhibits the hydrogenation reaction and C-C coupling in the catalyst. But the CO_2_ selectivity of the catalyst exceeds 50% at low CO conversion, which is mainly due to the direct dissociation and adsorption of O atoms in the CO dissociation process to form CO_2_ through CO* + O* → CO_2_ (* represents the adsorbed or activated species) and the other part of CO_2_ is generated by the WGSR. The synergetic effect can promote the selectivity of light olefins but it can also improve the selectivity of CO_2_ and promote the WGSR. When the C-C coupling is successfully suppressed, the C_5_^+^ hydrocarbon selectivity is low.

By reviewing different catalysts with Al_2_O_3_ as the support and Fe as the active component obtained by previous authors by changing the influencing factors and preparation methods, the results show that the highest yield of light olefins is about 27%, while CO_2_ selectivity is maintained at a high level (above 46%). Some typical data are shown in Table 2. The main reason is the joint effect of synergism and surface alkalinity, which makes the surface and nearby active Fe_x_C species have suitable adsorption and decomposition ability of CO and H_2_, and also have the appropriate desorption ability of light olefins species. SMSI, pore size, and synergistic effect have a promoting effect on the FTO reaction performance of the catalyst. With an understanding of the interaction between iron and the support, it was found that the interaction will inhibit the WGSR. By regulating the active components on the surface of the support and reducing the SMSI of the catalyst, the amount of the weakened medium and weak basic sites increased, which promotes the desorption of olefins and improves the selectivity of light olefins. The pore size has a significant confinement effect on the active components and products. The grain size and the distribution of the active components can be adjusted by the pore diameter, so as to adjust the amount of active sites. At the same time, the products generated on the surface of the active species will also be affected by steric hindrance, and a large pore size facilitates the formation of heavy hydrocarbons. The synergistic effect affects the adsorption and desorption ability of the reactants and products through the O atoms. In addition, the stability of the catalyst will also be affected by the support. The traditional support materials are mainly high-temperature-resistant oxides, such as Al_2_O_3_ and SiO_2_; a pure Fe catalyst has high FTO activity, but will slowly deactivate with time, while the CO conversion of an Fe/Al_2_O_3_ catalyst is stable, even when slowly increased. Obviously, the addition of oxides to the iron catalyst reduces the activity of the catalyst but improves the stability of the catalyst.

**Table 2:** Summary of FTO performance of iron catalysts supported by Al_2_O_3_.

Catalysts	Temperature (K)	Pressure (Mpa)	H_2_/CO	$X_{\mathrm{CO}}$ (%)	$S_{C_{n}}$ (%C mol)	$S_{{\mathrm{CO}}_{2}}$ (%)	Yield	Reference	Eqs.
Fe	533	1.5	0.7	75	---	45	---	[41]	---
Fe/Al_2_O_3_	533	1.5	0.7	19	---	28	---	[41]	---
Al_2_O_3_	618	1.5	2.0	72	---	49	---	[42]	(7) ^b^
50%MgO/Al_2_O_3_	618	1.5	1.0	91	30	46	27	[42]	(7) ^b^
MgO	618	1.5	1.0	74	---	46	---	[42]	(7) ^b^
S-Al_2_O_3_	633	0.1	---	16	24	38	4	[31]	(7) ^b^
M-Al_2_O_3_	633	0.1	---	12	24	41	3	[31]	(7) ^b^
L-Al_2_O_3_	633	0.1	---	12	25	42	3	[31]	(7) ^b^
5AFe	623	2.0	1.0	58	20	45	12	[45]	(7) ^b^
5AFe/S/Na	623	2.0	1.0	69	29	45	20	[45]	(7) ^b^
Fe-K_2_O/ZnAl_8_O_13_	633	2.0	1.0	15	30	54	5	[47]	(7) ^b^

^b^: Results are not based on 1 g Fe, ---: No original data.

## 4. Molecular Sieve

The molecular sieve is a new material with a large specific surface area (>1000 m^2^/g), a three-dimensional pore structure, a pore size ranging from 2 nm to 50 nm, and a single pore size distribution. Therefore, it has an excellent shape selection effect on reactants, products, etc. The surface adsorption capacity is high, the hydrophobic property is adjustable, and the skeleton contains activation centers such as acid centers, so it is widely used as an FTO support.

### 4.1. Acid Regulation

A ZSM-5 molecular sieve has acidic sites. Weak acidity will inhibit the secondary hydrogenation of olefins, improve the CO conversion and stability of the catalyst, and improve the selectivity of light olefins [49]. However, a high acid catalyst makes it easy to cause secondary cracking, resulting in excessive cracking [50], and therefore its sensitivity to the reaction temperature is too high, which makes it easy to coke and deactivate [51].

**Figure 3:**
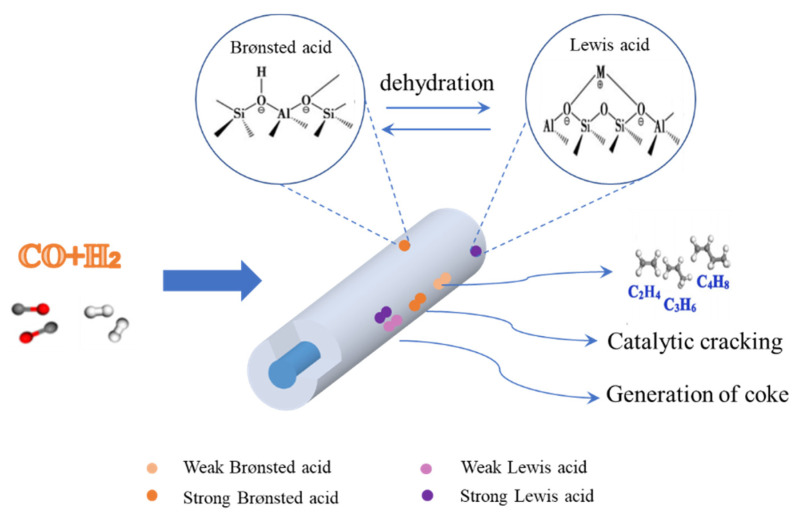
The influence diagram of Brønsted and Lewis acid on the reaction performance of the catalyst.

Kang et al. [52] studied the effect of different types of molecular sieves as carriers (ZSM-5, Mordenite, and Beta-zeolite) on the reaction performance of Fe-Cu-K catalysts, as shown in Table 3. It was found that the conversion of CO on Fe-Cu-K/ZSM-5 is the highest (81%), mainly because the ZSM-5 molecular sieve retains an original three-dimensional pore structure after calcination, which can promote the reduction of active components and form more active species. The acid strength of the three zeolites is: Fe-Cu-K/ZSM-5 > Fe-Cu-K/Beta-zeolite > Fe-Cu-K/Mordenite. The total number of acid sites varies in the order of Fe-Cu-K/Mordenite > Fe-Cu-K/Beta-zeolite > Fe-Cu-K/ZSM-5. The Fe-Cu-K/Beta-zeolite catalyst has a higher selectivity of light olefins (22%). The reason for this phenomenon is the strength and number of acid sites on the surface of the catalyst. The surface of Fe-Cu-K/Beta-zeolite has a lower acid strength and a higher acid site density than the other two types of supports. Therefore, the intermediate olefins generated by the reaction are not favorable to secondary hydrogenation and are more easily desorbed from the surface of the active species, which leads to an increase in olefin selectivity. Mordenite has a two-site pore structure and is easily deactivated during the catalyst reaction. Due to the uneven acid distribution of the ZSM-5 molecular sieve, the light olefins produced by the reaction make it easy to continue the hydrogen transfer and other reactions to generate alkane (ethane, propane, butane, etc.) and coke under the action of the strong acid center of the catalyst. The selectivity of the target product is reduced. Therefore, the strength of Brønsted acid can be weakened by decreasing the Si/Al ratio, which can inhibit the hydrogen transfer and improve the selectivity of light olefins [53]. Kang et al. [54] studied the effect of Fe-Cu-K catalysts supported with a ZSM-5 with three different Si/Al ratios on the reaction performance, as shown in Table 3 (Si/Al = 25, Si/Al = 40, and Si/Al = 140). The composite catalyst prepared using the ZSM-5 with a Si/Al ratio of 25 has a higher CO conversion (81%) and light olefin selectivity (6%). PlanaPalleja et al. [55] also found that a low Si/Al ratio will increase the selectivity of light olefins. A decrease in the Si/Al ratio will increase the density of the Brønsted acid sites, and then reduce the acid strength of the acid sites. The decrease in acid strength will reduce the probability of the secondary hydrogenation of olefins and increase the selectivity of light olefins, as shown in Figure 3. The acid–alkali properties of catalysts are mainly divided into two categories: Brønsted acid–alkali (Brønsted acid, Brønsted alkali) and Lewis acid–alkali (Lewis acid, Lewis alkali). The most basic structural units of zeolite molecular sieves are silicon–oxygen and aluminum–oxygen tetrahedrons. The silicon–oxygen tetrahedron is neutral, and in the aluminum–oxygen tetrahedron [(AlO_4_)^−^], because aluminum is +3 valence, the tetrahedron is negatively charged. The Brønsted acid center and Lewis acid center usually coexist on acidic molecular sieves. It is generally accepted that two Brønsted acids form a Lewis acid after removing structural water above 500 °C, and this conversion process is reversible. The pure silica molecular sieve does not have any acidity. When Al^3+^ replaces Si^4+^ and is introduced into the molecular sieve skeleton, the electrical neutrality of the overall structure is broken, and additional cations are required to balance the negative charge of the skeleton. When the cation is H^+^, the molecular sieve has acidity, so the position of the Al atom in the molecular sieve directly determines the specific distribution of the Brønsted acid sites on the molecular sieve skeleton, which in turn determines the catalytic CO conversion and selectivity. In addition, the specific position of the Al on the molecular sieve framework can also affect the local environment of the acid site, the accessibility of the acid site, and the acid strength. This is mainly because the T-O-T (T represents Si, Al, or P) angle and T–O bond length of the corresponding skeleton tetrahedron will change when the aluminum atom replaces the skeleton silicon atom. It is generally believed that the higher the Al content, the smaller the Al-O-Si angle and the weaker the acid strength [55].

### 4.2. Structure

**Figure 4:**
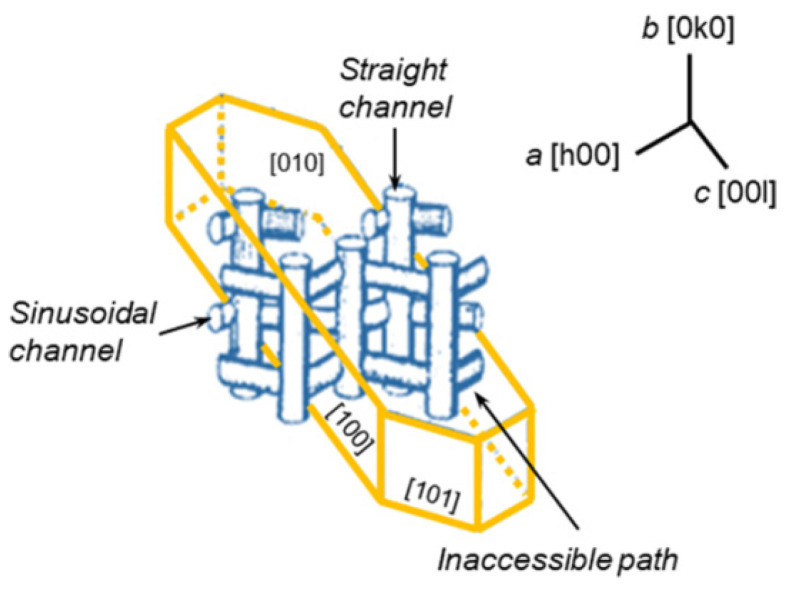
Projections of the channel directions and orientations in ZSM-5 crystallite [58].

A molecular sieve has unique shape selectivity, which can improve the selectivity of light olefins. The pore structure of the ZSM-5 is formed by the intersection of straight pores and ‘Z’ shaped pores. Pentasil zeolite with a 10-membered ring opening has an ordered pore structure and molecular shape selectivity produced by a unique skeleton structure [56,57]. As shown in Figure 4, there is a set of sinusoidal channels with a pore size of 0.51 × 0.51 nm perpendicular to the (100) plane, and a set of straight channels with a pore size of 0.51 × 0.56 nm perpendicular to the (010) plane, and the two sets of channels cross each other to form a larger cross cavity [58].

When the pore size of the molecular sieve is small (the molecular size of the light olefin is smaller than the pore size of the molecular sieve), the light olefin will go out directly from the pore. When the molecular size of C_5_^+^ is larger than the pore size of the molecular sieve, it cannot be directly removed, and it will continue to crack into light olefins in the pores and then diffuse outside of the pores of the molecular sieve, resulting in an increase in the selectivity of light olefins [59]. For molecular sieves with high acidic site strength (low Al content), the outer surface of the molecular sieves is generally an important place for catalytic reactions. The reason for this may be due to the rapid coking of the internal channel. Therefore, in order to make the catalytic reaction proceed inside the molecular sieve and improve the selectivity of light olefins by shape selectivity, it is necessary to reduce the strength of the acid sites outside the molecular sieve. Kang et al. [54] investigated the effects of three Si/Al ratios (Si/Al = 25, Si/Al = 40, Si/Al = 140) on the acid site strength, density, and light olefin selectivity, and found that the Si/Al ratio is positively correlated with the acid site strength, but negatively correlated with the acid site density and light olefin selectivity.

A new type of core–shell catalyst can also be designed in which the core catalyst is tightly packed with a zeolite shell, providing a customized closed reaction environment that results in the space-limiting effect and shape-selective function of the FTO reaction, contributing to the formation of light olefins and inhibiting the formation of undesirable long-chain hydrocarbons. Jiang et al. [60] covered a layer of silicalite-1 zeolite shell on the Fe/SiO_2_ catalyst using secondary synthesis, as shown in Table 3 (Fe/SiO_2_ and Fe/SiO_2_-S). The CO conversion of the Fe/SiO_2_-S catalyst (21%) is lower than that of the Fe/SiO_2_ catalyst (41%). The main reason for this is that the secondary synthesis process will cause some damage to the Fe/SiO_2_ catalyst or form some zeolite crystals covering part of the active species, thereby reducing the conversion of CO. However, the light olefin selectivity of the Fe/SiO_2_-S catalyst (30%) is higher than that of the Fe/SiO_2_ catalyst (20%). This is due to the use of the space limitation and separation function of the silicalite-1 zeolite shell; the light olefin produced by the catalyst is rapidly diffused outside the catalyst.

**Figure 5:**
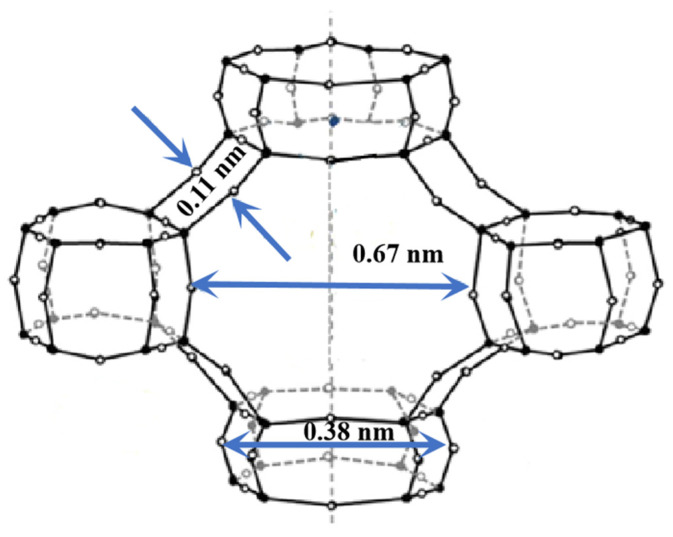
Skeleton structure diagram and pore size of the unit cell of SAPO-34. Each solid circle represents an Al, P, or Si and the open circles represent oxygen [61].

SAPO-34 (silicon aluminum phosphate molecular sieve) is a kind of small pore molecular sieve (pore diameter: 0.38 nm) with a CHA framework. The pore structure is composed of interconnected large cages of 0.67 × 0.11 nm and small pores of 0.38 × 0.38 nm (8-MR), as shown in Figure 5 [61,62].

**Figure 6:**
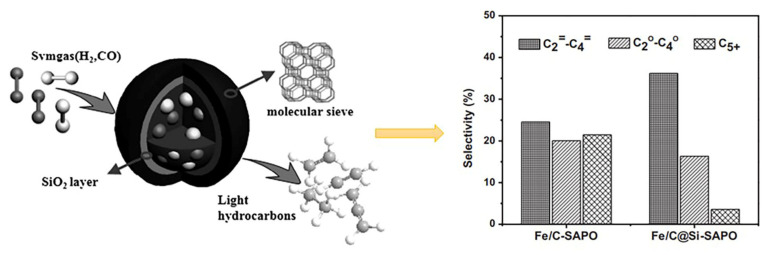
The schematic representation and hydrocarbon distributions of Fischer–Tropsch synthesis on core–shell structured catalyst [63].

It can also be used as a shell structure to achieve spatial confinement. As shown in Figure 6, during the FTO reaction, CO (kinetic diameter: 0.376 nm) and H_2_ (kinetic diameter: 0.289 nm) can enter the core region through the shell channels (pore diameter: 0.38~0.43 nm) of the catalyst prepared with SAPO-34 as the shell. A wide range of hydrocarbons are generated in the core region, and small molecular hydrocarbons (kinetic diameter less than the pore) can pass through the shell. Macromolecular hydrocarbons (kinetic diameters greater than the pores) need to continue to undergo cracking reactions in the pores to convert into small molecular hydrocarbons that can pass through the shell or undergo hydrogen transfer or condensation reactions to form carbon deposits [63].

### 4.3. Metal Modification

Compared with Al-doped molecular sieves, the ion exchange capacity of pure silica molecular sieves is weak, and the acid content and acid strength are low, so its reactivity is low. It is necessary to introduce metal elements into the framework of the molecular sieve for modification, increase the number of defects in the framework, and thus improve the CO conversion. Through the introduction of exchangeable metal ions, the acidity, basicity, and surface adsorption properties of molecular sieves are improved, the hydrothermal stability is greatly improved, and the WGSR is reduced.

K is an alkali metal electronic promoter, which can change the adsorption of reactants on the active site. This alkali metal promoter can enhance the chemical adsorption of CO because Fe can easily extract electron density from alkali metals, increasing the strength of the Fe–CO bond and weakening the C–O bond in the molecule. At the same time, higher alkalinity will also lead to a decrease in weakly bound H_2_ on the catalyst surface, reducing the hydrogenation activity, increasing the selectivity of light olefins, and reducing the selectivity of methane [64,65]. Guo et al. [66] studied the effect of K on the reaction performance of the Fe/NaY catalyst. It was found that the addition of K can provide electrons to the active component Fe and increase its electron cloud density, thereby promoting the dissociative and adsorption of CO, increasing the CO conversion of the catalyst (Fe/NaY: 57% → 10Fe-2K/NaY: 78%), inhibiting the adsorption of H_2_, reducing the secondary hydrogenation of light olefins, and thus promoting the formation of light olefins (Fe/NaY: 5% → 10Fe-2K/NaY: 37%).

**Figure 7:**
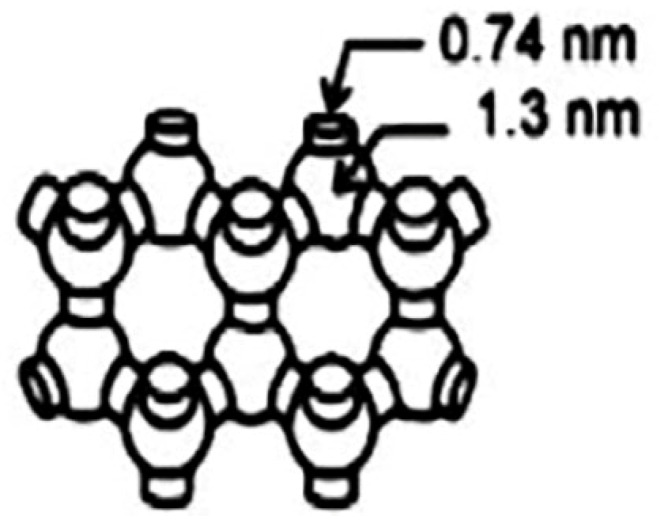
Diagram of pore diameter of Y zeolite.

Y zeolite is a microporous molecular sieve with a three–dimensional pore structure, due to its high specific surface area, large cavity volume (pore diameter is 0.74 nm), and suitable acid properties [67,68,69], as shown in Figure 7. Wang et al. [70] investigated the effect of loading different alkali metal promoters on Fe/Y catalysts for an FTO reaction, as shown in Table 3 (Fe-Li/Y, Fe-Na/Y, Fe-K/Y, and Fe-Cs/Y). Among them, alkali metal ions are loaded on zeolite Y for modification, with the atomic number of alkali metal ions increasing gradually from Li^+^ to Cs^+^, the surface basicity of the catalyst is also gradually increased, and the CO dissociation rate is greatly improved. This shows that an increase in the atomic number will promote the adsorption and desorption of CO on the catalyst surface and improve the formation of the active species; the CO conversion also increased from 49% to 91%. At the same time, the selectivity of CO_2_ also increases significantly from 9% to 41%, which is mainly due to the electronic effects of the alkali metal promoters accelerating the WGSR. Alkali metal atoms are positively charged, while O is negatively charged. As the atomic number increases, the attraction of the nucleus to the extranuclear electrons gradually increases. Strong electrostatic attraction between positive and negative charges will make the adsorption of oxygen-containing species more stable, thus reducing the dissociation energy barrier and increasing the selectivity of CO_2_. The Lewis alkali strength of the alkali metal promoters increases with the increase in atomic number; some strong acid sites (Brønsted acid) on the molecular sieve are converted into weak acid sites (Brønsted acid), reducing the secondary adsorption probability of olefins, thereby improving the selectivity of light olefins.

**Table 3:** Summary of FTO performance of iron catalysts supported on molecular sieve.

Catalysts	Temperature (K)	Pressure (MPa)	H_2_/CO	$X_{\mathrm{CO}}$ (%)	$S_{C_{n}}$ (%C mol)	$S_{{\mathrm{CO}}_{2}}$ (%)	Yield	Reference	Eqs.
Fe-Cu-K/Mordenite	573	2.0	1.0	73	12	38	9	[52]	(7) ^b^
Fe-Cu-K/Beta-zeolite	573	2.0	1.0	64	22	25	14	[52]	(7) ^b^
Fe-Cu-K/ZSM-5	573	2.0	1.0	81	20	36	16	[52]	(7) ^b^
(Si/Al = 25)	573	1.0	2.0	81	6	38	5	[51]	(7) ^b^
(Si/Al = 40)	573	1.0	2.0	79	6	37	4	[54]	(7) ^b^
(Si/Al = 140)	573	1.0	2.0	62	3	29	2	[54]	(7) ^b^
Fe-SiO_2_	653	1.0	0.5	41	20	---	8	[60]	(4) ^b^
Fe/SiO_2_-S	653	1.0	0.5	21	30	---	6	[60]	(4) ^b^
Fe/NaY	593	2.0	2.0	57	5	38	3	[66]	(7) ^b^
Fe-K/NaY	593	2.0	2.0	78	37	41	29	[66]	(7) ^b^
Fe/LiY	543	2.0	2.0	40	---	9	---	[70]	---
Fe/NaY	543	2.0	2.0	49	---	21	---	[70]	---
Fe/KY	543	2.0	2.0	75	---	36	---	[70]	---
Fe/CsY	543	2.0	2.0	91	---	41	---	[70]	---

^b^: Results are not based on 1 g Fe, ---: No original data.

The acidity and shape selectivity of molecular sieves and electron promoters have a remarkable effect on the reaction performance of the catalyst. Reducing the Si/Al ratio will create more weakly acidic sites, reduce the probability of secondary hydrogenation of olefins, inhibit the occurrence of the hydrogen transfer reaction of olefins, and improve the selectivity of light olefins. The unique three-dimensional pore structure of the molecular sieve has a space limitation effect and molecular shape selectivity, which will improve the selectivity of light olefins. The addition of alkali metal promoters will increase the pore size of the molecular sieve, creating timely diffusion of olefins. Secondly, if the acidity of the catalyst is reduced, the probability of secondary adsorption of olefins is reduced, and the selectivity of light olefins is increased.

## 5. Carbon Material

**Figure 8:**
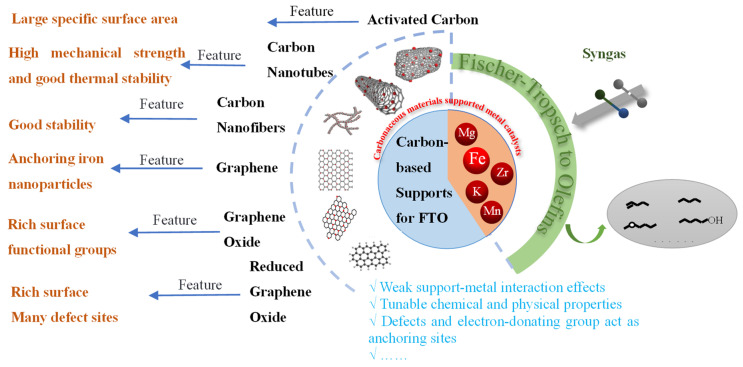
Carbonaceous support materials [75].

Carbon materials are ideal supports with considerable specific surface areas and adjustable pore structures. They can effectively improve the dispersion and electronic properties of the active components and ensure that the catalyst has good mechanical properties. Compared with metal oxides, first, the interaction with the active component Fe is weaker. In some cases, this interaction will be in a controllable stage, thereby adjusting the surface properties of the catalyst and improving its reaction performance. Second, their cost is low and has a high industrial application prospect. Therefore, in recent years, activated carbon (AC), carbon nanotubes (CNTs), graphene (GN), graphene oxide (GO), and reduced graphene oxide (rGO) have been widely studied as supports of Fe-based catalysts for an FTO reaction, as shown in Figure 8 [71,72,73,74,75].

**Figure 9:**
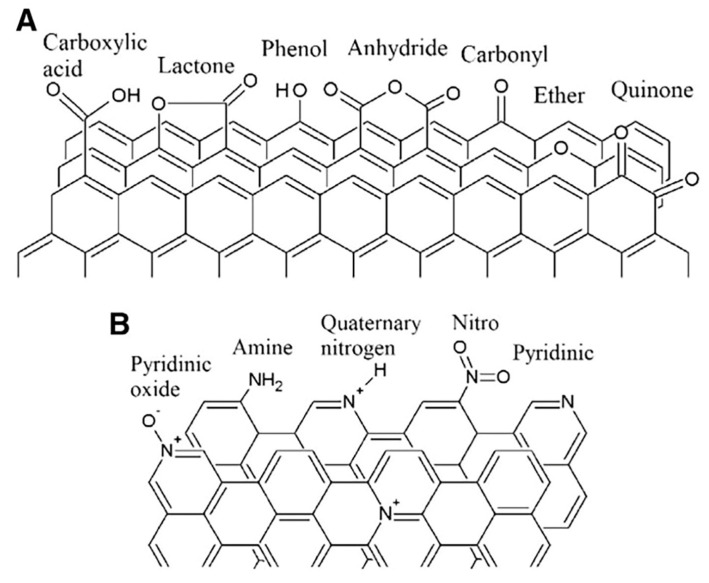
Carbon surface oxygen (**A**) and nitrogen functional groups (**B**) [76].

Carbon materials have excellent properties but their chemical inertness and hydrophobic surface cause sintering and agglomeration of active components and poor stability. Therefore, new valence bonds and oxygen or nitrogen functional groups are formed on the carbon surface by changing the content of elements on the carbon surface or under different reaction conditions, as shown in Figure 9. The surface morphology, structure, and chemical composition of the catalyst are affected differently, and the reaction performance is also affected [76].

### 5.1. Activated Carbon

Activated carbon is widely used because of its large pore structure, high specific surface area, and rich functional groups.

The addition of alkali metal promoters and transition metal promoters will promote the dissociative and adsorption of CO and improve CO conversion and light olefin selectivity. An alkali metal promoter can provide electrons to Fe, strengthen the C–Fe bond, weaken the C–O bond, promote the dissociation and adsorption of CO, increase the concentration of active species Fe_x_C, and increase the conversion of CO. The transition metal and alkali metal promoters have similar effects. Both of them promote the dissociation and adsorption of CO through electron loss, inhibit the adsorption of H_2_, reduce the secondary hydrogenation of olefins, and increase the selectivity of light olefins. Chernavskii et al. [77] studied the effect of the alkali metal promoter K on the reaction performance of an Fe/AC catalyst. As shown in Table S1, the following two catalysts are prepared: K-Fe/AC and Fe/AC. Among them, K-Fe/AC refers to the first impregnation with KNO_3_ and then impregnation with Fe(NO_3_)_3_. It was found that the addition of K increased the CO conversion of the catalyst (Fe/AC: 32% → K-Fe/AC: 62%). The main reason is that the K promotes CO dissociation through electron transfer to form an active species (*χ*-Fe_5_C_2_), thereby improving the CO conversion of the catalyst. Compared with the Fe/AC catalyst, the content of Fe_3_O_4_ in the K-Fe/AC catalyst increased from 55% to 61%. The main reason is that the number of oxygen-containing groups on the surface of AC increases during the reaction. These groups interact with the Fe^3+^ ions to form more nucleation centers and more Fe_3_O_4_, which promotes the WGSR. Asami et al. [78] studied the effect of Mn on the reaction performance of the Fe-Cu/AC catalyst. It was found that Mn^2+^ is evenly distributed on the surface of AC and formed complex oxides with iron oxide, which reduces the adsorption capacity of hydrogen, resulting in a decrease in hydrogen surface concentration and increased the selectivity of light olefins (Fe-Cu/AC: 25% → Fe-Cu-Mn/AC: 33%).

As a strong oxidant, KMnO_4_ can not only increase the oxygen-containing functional groups and surface defects on the surface of the carbon materials but also form a K-doped MnO_x_ coating on the surface of the carbon materials. Tian et al. [79] improved the selectivity of light olefins by preparing a KMnO_4_-modified Fe/AC catalyst (Fe-AC: 7% and Fe-2MnK-AC: 15%). It was found that KMnO_4_ can be used as an oxidant, namely 4MnO_4_^−^ + 3C + H_2_O → 4MnO_2_ + CO_3_^2−^ + 2HCO_3_^−^. It causes the surface of AC [80] to have more oxygen-containing groups and defect sites, which is helpful to anchor the α-Fe_2_O_3_ particles. Secondly, MnO_2_ is converted into Mn_3_O_4_ by calcination, which promotes the reduction and formation of active sites, reduces the secondary hydrogenation reaction of olefins, and improves the selectivity of light olefins. In addition, K promoters will promote the formation of carbides, improve the CO conversion (Fe/AC:62% → Fe-2MnK-AC:97%), and greatly increase the yield of light olefins (Fe/AC: 4% → Fe-2MnK-AC: 15%).

The electronic properties of carbon supports can be modulated by using electron-rich atoms, such as nitrogen atoms, so that electrons can be transferred from the electron-rich atoms to the metal species to alter the catalytic performance. Tian et al. [81] studied the effect of N doping on the reaction performance of an Fe-KMnO_4_/AC catalyst. On one hand, N atoms partially replace O atoms and enter into the Fe lattice to form iron nitride [82,83], which reduces CO_2_ selectivity from 62% (Fe-AC) to 47% (FeN-10MnK-AC), and inhibits the WGSR, as shown in Table S1. On the other hand, the introduced N atoms change the electronic environment around the active sites. Electron pairs of nitrogen atoms occupy the electron hole pairs of the adsorbed H^+^ ions. This retards the formation of the C–H bond and ulteriorly limits the second hydrogenation of unsaturated hydrocarbons adsorbed on the active sites, increasing the selectivity of light olefins (Fe/AC: 9% → FeN-10MnK-AC: 19%).

### 5.2. Carbon Nanotubes (CNTs)

Carbon nanotubes (CNTs) have a large specific surface area, high thermal conductivity, and stability. Therefore, they become a new catalyst support with great application potential.

CNTs are radial structures with hollow interiors formed by curling graphene sheets, typically with an internal and external diameter of between 5~30 nm and 8~60 nm, respectively. Their internal electron density will flow outward, resulting in a small internal electron density and many defect sites. Due to their unique hollow structure, they easily react with active components and improve CO conversion [84]. Chen et al. [72] showed that the conversion of CO in an Fe-in-CNT catalyst (40%) is higher than that of an Fe-out-CNT catalyst (29%). The main reason is that the electron density inside the CNTs is lower than that on the outside. Therefore, when the active component enters the interior from the outside, it becomes unstable which makes it easier to reduce due to the internal electron density being smaller than the external. Abbaslou et al. [85] also found that active components on the inner surface of carbon nanotubes are more likely to form active species, and the CO conversion is higher after 125 h (in-Fe/CNTs: 85% and out-Fe/CNTs: 79%, as shown in Table S1).

Due to their inert surface, carbon nanotubes must be functionalized or modified in the liquid or gas phase. Acid treatment can effectively remove the amorphous carbon in the carbon nanotube, improve the dispersion of carbon nanotubes; at this time the introduction of electronic promoters can promote CO conversion. KMnO_4_ usually produces a large amount of -COOH groups on carbon nanotubes, which can be removed at 553 K, resulting in more defects. These defects will promote the transfer of electrons from the active component to the support, thereby allowing the reactant to adsorb and stabilize on the nanoparticles, which are supported on the carbon nanotubes, and thus improving the reaction performance of the catalyst. N-doped carbon nanotubes (NCNTs) can provide effective anchoring points to facilitate the immobilization of highly dispersed metal nanoparticles.

Fang et al. [86] studied the effect of a Mn promoter on the catalytic performance of Fe/CNTs after acid treatment. It was found that after the acid treatment of the CNTs, the CO conversion increases (Fe/CNTs: 30% → Fe/CNTs-D: 40%). The addition of a Mn promoter reduces the particle size of the active component, promotes the reduction and carbonization of the catalyst, and further improves the CO conversion (Fe/CNTs-D: 40% → FeMn5/CNTs-D: 42%), as shown in Table S1. Wang et al. [87] impregnated Fe on MnK-CNTs by modifying CNTs with KMnO_4_, as shown in Table S1 (Fe/MnK-CNTs). It was found that the CO conversion was 23%. The main reason for this was that the defect sites formed by the KMnO_4_ oxidation will stabilize the FeMnK-containing nanoparticles on the carbon nanotubes, which are highly dispersed and uniformly distributed on the carbon nanotubes, facilitating the reduction and carbonization of the active components. Moreover, Mn^2+^ will be converted to Mn_3_O_4_ composite oxides, the adsorption and dissociation of H_2_ active sites are inhibited, the secondary hydrogenation of olefins is reduced, and the selectivity of light olefins is increased (32%). Lu et al. [88] studied the effect of N doping on the reaction performance of iron-based catalysts, as shown in Table S1 (Fe/CNTs and Fe/NCNTs). It was found that the CO conversion of the catalyst increases (Fe/CNTs: 9% → Fe/NCNTs: 14%) and the selectivity of light olefins increases (Fe/CNTs: 30% → Fe/NCNTs: 38%). Due to the inherent alkalinity and anchoring effect of the N-doped carbon nanotubes, Fe can be immobilized on them without any acid–alkali modification so that electrons can be transferred from the nitrogen to the iron [89], thereby increasing the electron density of the iron, enhancing the Fe–C bond, weakening the C–O bond, enhancing CO adsorption and dissociation, inhibiting H_2_ adsorption, and promoting the selectivity of light olefins. The Fe/NCNT catalyst can also promote a reduction in the active component and the formation of the active species *χ*-Fe_5_C_2_, and improve the CO conversion of the catalyst. Bao et al. [90] studied the CO hydrogenation reaction of the N-doped carbon nanotubes. It was found that the CO conversion of an N-doped catalyst is eight-times higher than that of a non N-doped catalyst. The main reason is that N doping will cause the formation of Fe_x_N; therefore, the catalyst has better oxidation resistance and is not easy to water oxidize, which can lead to deactivation.

### 5.3. Graphene (GN), Graphene Oxide (GO), and Reduced Graphene Oxide (rGO)

Graphene is a two-dimensional carbon nanomaterial with a hexagonal honeycomb lattice (similar to a benzene ring structure) composed of sp2 hybrid orbitals of carbon atoms. Its’ structural characteristics are called Mobius ring topological characteristics. Graphene is the hardest and thinnest new material in the world, and it also has the advantages of high strength and high specific surface area [91,92,93,94]. Compared with other carbon materials, graphene has a perfect hybrid structure; a large conjugated system makes its’ electron transport capacity strong with excellent electrical conductivity. However, graphene has a high radius–thickness ratio and there is a high van der Waals force between the layers. The interaction between the π–π bonds is weak, making it prone to agglomeration [95]. It can hardly be uniformly dispersed in water and organic solvents by chemical bonds, nor can it be stably combined with active components. Graphene oxide and graphene are two-dimensional carbon nanostructures. The molecular structure of graphene oxide is to introduce oxygen-containing functional groups on the surface of the molecular structure of graphene. These oxygen-containing functional groups will make the surface of the graphene oxide hydrophilic, thereby increasing the interaction force between the π–π bonds, overcoming the van der Waals force between the layers, enhancing its dispersibility in water, and introducing other functional groups for graphene oxide. The reaction provides the best binding site, making the application of graphene oxide more extensive [96,97,98]. However, the molecular structure of graphene is destroyed to a certain extent. Although the defect sites are increased, the conductivity and mechanical properties of the graphene oxide will deteriorate [99]. Reduced graphene oxide is obtained by reducing graphene oxide to reduce the oxygen-containing functional groups on the surface. The reduction in the oxygen-containing functional groups will increase the π-conjugated structure and increase the conductivity. At the same time, the reduced graphene oxide has serious damage to the skeleton structure and a higher number of defect sites due to the oxidation and reduction operations. The abundant defect sites favor the loading of promoters on the catalyst and promote the reduction and carbonization of the active components [100]. Cheng et al. [101] studied the addition of Mg and K to an Fe/rGO (reduced graphene oxide) catalyst, as shown in Table S1 (FeMnK1/rGO). It was found that Mg can effectively inhibit the formation of Fe_3_O_4_, inhibit the WGSR, and reduce the selectivity of CO_2_. Its biggest disadvantage is that the CO conversion of the catalyst and the selectivity of light olefins are low. K can promote the active species, effectively inhibit the generation of CH_4_, and inhibit the secondary hydrogenation reaction of light olefins, making up for this shortcoming. Therefore, the synergistic effect of the Mg and K promoter can not only inhibit the WGSR, but also promote the dissociation and adsorption of CO, and increase the selectivity of light olefins (22%). Cheng et al. [102] studied the effect of K on the reaction performance of the Fe/rGO catalyst in the FTO reaction, as shown in Table S1 (Fe/rGO, FeK1/rGO, and FeK2/rGO). It was found that K directly acted on the graphene oxide to modify it. When K = 2wt%, it was found that K can effectively inhibit the methane production and the secondary hydrogenation of olefins, and the selectivity of light olefins reaches the maximum (27%).

**Figure 10:**
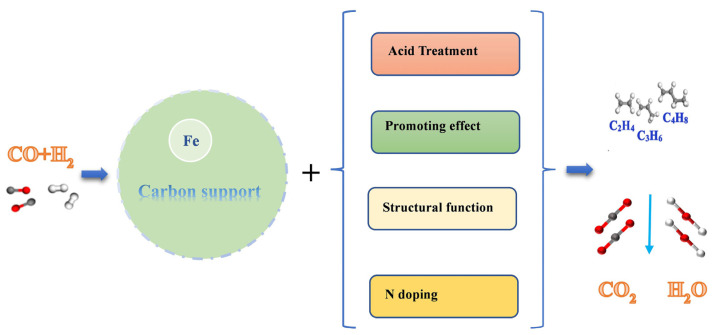
Influencing factors and reaction properties of carbon-based materials.

As shown in Figure 10, carbon materials can be modified by adding promoters, changing pore size, adjusting surface acidity and alkalinity, and doping heteroatoms (such as O, N atoms, and so on), so as to change the dispersion, stability, reduction, and carbonization properties of the active components, change the support limited effect, and finally achieve the adjustment of the catalyst reaction performance. Higher CO conversion and light olefin selectivity, lower CO_2_ selectivity, light alkanes and C_5_^+^ selectivity are the direction and purpose of the catalyst reaction performance regulation. By adding promoters and surface heteroatoms (such as KMnO_4_, HNO_3,_ and N doping) to carbon materials, the surface electrons, acid–alkali, and anchoring effects of the supports and promoters on the active components can be changed, so as to change the reaction performance of the catalysts. The promoters or some surface heteroatoms will act as electron donors to provide electrons to the Fe, strengthening the C–Fe bonds, weakening the C–O bonds, promoting the dissociation and adsorption of CO, and reducing the adsorption and dissociation of the H_2_ active species, resulting in a decrease in the hydrogenation performance of the active species, which in turn hinders the secondary hydrogenation reaction of olefins and improves the selectivity of light olefins. The introduction of some heteroatoms will change the acidity and alkalinity of the catalyst and/or produce a large number of defect sites and oxygen-containing functional groups. The enrichment of defects and oxygen-containing groups anchors the *α*-Fe_2_O_3_ particles with a smaller average particle size, avoids agglomeration and sintering, increases the dispersion of active components, and improves CO conversion.

## 6. Hydrophobicity and Amphiphobic Modification

### 6.1. Hydrophobic Modification of Metallic Oxide

**Figure 11:**
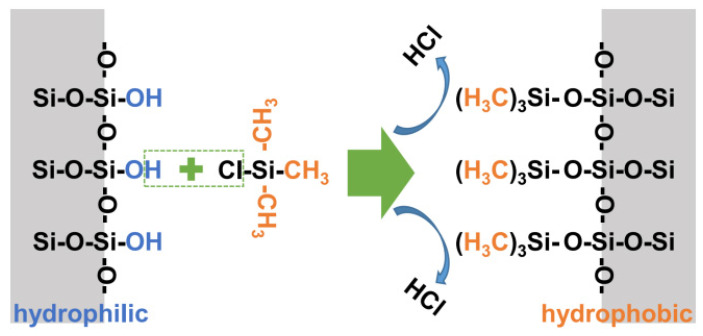
Hydrophobic modification through silanization reaction [103].

The hydrophobicity of the catalyst surface will change the adsorption behavior of the FTO products, such as light olefins and water, affecting the distribution of the products. In recent years, organic–inorganic hybrid materials have been widely studied and great progress has been made in many fields, including the catalytic field. Firstly, hydrophobic modification can inhibit this interaction force, reduce the difficulty of O^2−^ removal, and increase reduction and carbonization. Secondly, hydrophobic modification makes water desorb from the catalyst surface in time, inhibits the WGSR, and reduces CO_2_ selectivity. Metal oxides such as SiO_2_ and Al_2_O_3_ have -OH on their surfaces, and -OH are common hydrophilic groups that absorb more H_2_O. The hydrophilic groups are generally -OH, -COOH, -CHO and the hydrophobic groups are generally -CH_3_, -(CH_3_)_2_, -(CH_3_)_3_, etc. Hydrophobic modification is achieved by replacing the hydroxyl groups on the catalyst support with hydrophobic groups, thereby achieving the purpose of changing the surface properties, as shown in Figure 11 [103].

**Figure 12:**
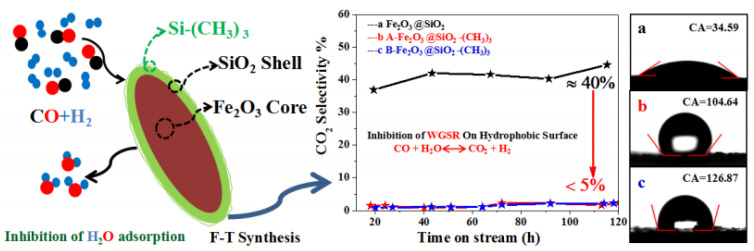
Schematic diagram of structure and WGSR and water contact angles of (**a**) Fe_2_O_3_@SiO_2_, (**b**) A-Fe_2_O_3_@SiO_2_-(CH_3_)_3_, and (**c**) B-Fe_2_O_3_@SiO_2_-(CH_3_)_3_ sample [11]. Stars stand for CO_2_ selectivity.

There is an interaction force between SiO_2_ and Fe, which will reduce the reaction performance of the catalyst, while hydrophobic modification can change the surface properties and promote the reaction performance of the catalyst. It will reduce the concentration of H_2_, inhibit the secondary hydrogenation reaction of olefins, and improve the selectivity of light olefins [36]. Yu et al. [11] prepared the core–shell Fe_2_O_3_@SiO_2_ catalyst modified using a methyl group (TMCS), as shown in Table 4. Fe_2_O_3_@SiO_2_, A-Fe_2_O_3_@SiO_2_-(CH_3_)_3_, B-Fe_2_O_3_@SiO_2_-(CH_3_)_3_, respectively, represent no methyl modification, adding a one-times methyl modification, and adding a two-times methyl modification. It was found that the contact angles of Fe_2_O_3_@SiO_2_, A-Fe_2_O_3_@SiO_2_-(CH_3_)_3_, and B-Fe_2_O_3_@SiO_2_-(CH_3_)_3_ are 34.59°, 104.64° and 126.87°, respectively, and they have obvious hydrophobicity on the surface, which causes water to desorb from the catalyst surface in time, thus inhibiting the WGSR. At the same time, the A-Fe_2_O_3_@SiO_2_-CH_3_ catalyst has good stability under the reaction conditions of 593 K, 1.5 Mpa, and H_2_/CO = 2, and the CO_2_ selectivity is only 5%, as shown in Figure 12. Moreover, the light olefin selectivity of the A-Fe_2_O_3_@SiO_2_-CH_3_ catalyst (20%) is higher than that of the Fe_2_O_3_@SiO_2_ catalyst (8%). The main reason for this is that the decrease in the WGSR does not cause the increase in partial pressure of hydrogen near the active sites, thus inhibiting the secondary hydrogenation reaction of olefins, and leading to the improvement of the selectivity of light olefins. When the catalyst changes from hydrophilicity to hydrophobicity, its strong hydrophobicity can promote the reduction of Fe_2_O_3_ on the surface, inhibit the WGSR, and provide more active sites for the FTO reaction [104]. However, it was found that the CO conversion is negatively correlated with the surface hydrophobic groups, that is, the CO conversion of Fe_2_O_3_@SiO_2_, A-Fe_2_O_3_@SiO_2_-(CH_3_)_3,_ and B-Fe_2_O_3_@SiO_2_-(CH_3_)_3_ are 96%, 54%, and 50%, respectively. The reason for this is that the more silane reagents, the more active sites will be covered by silane reagents, resulting in fewer active sites for the reactants to access, and therefore, lower the CO conversion. Li et al. [12] discussed the influence of different silica shell contents on the reaction performance of hydrophobic FeMnO_x_@SiO_2_-y-(CH_3_)_3_ (y represents the weight percentage of silica in the catalyst), as shown in Table 4 (FeMnO_x_, FeMnO_x_@SiO_2_-8.2-(CH_3_)_3,_ and FeMnO_x_@SiO_2_-13.1-(CH_3_)_3_). When the SiO_2_ content is 8.2%, the CO_2_ selectivity is only 5%. In this case, the selectivity of light olefins and the CO conversion of the catalyst can reach 55% and 51%, respectively. The separation of water from the active site during the FTO reaction is affected by two aspects. On the one hand is the number of hydrophobic functional groups, on the other hand is the thickness of the hydrophobic coating. When the content of the hydrophobic silica coating is lower than 8.2%, the number of hydrophobic functional groups plays a dominant role. Therefore, with the increase in the content of the hydrophobic silica coating, the water generated by the reaction can leave the active site and pass through the hydrophobic silica coating in time, which inhibits the WGSR and leads to the gradual decrease in CO_2_ content. However, when the content of the hydrophobic silica coating is higher than 8.2%, the thickness of the hydrophobic silica coating plays a dominant role. Therefore, with the increase in the content of the hydrophobic silica coating, the resistance of the generated water passing through the coating becomes greater, which restricts the rapid discharge of water, and thus increases the partial pressure of water near the active site, resulting in the oxidation of the active component [37]. The WGSR is promoted, and CO_2_ selectivity is improved.

Compared with SiO_2_, the basic reason why the Fe/Al_2_O_3_ catalyst is not hydrophobically modified is that most of the hydrophobic agents mainly contain Si ions. When SiO_2_ is used as the support, no other impurity ions are doped. When Al_2_O_3_ is used as the support, the doped Si ions will affect the properties of the catalyst and it is impossible to accurately determine which party determines the change in catalyst performance. Therefore, the Fe/Al_2_O_3_ catalyst is not suitable for hydrophobic modification.

### 6.2. Hydrophobic Modification of the Molecular Sieve

Through alkyl modification of the molecular sieve, the catalyst surface becomes hydrophobic, and then the WGSR is controlled.

**Figure 13:**
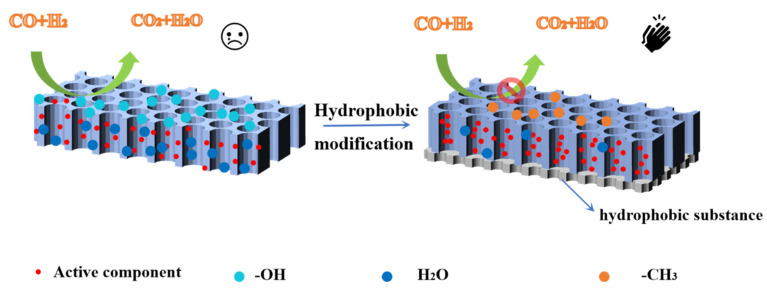
Hydrophobic modification principle diagram.

The hydrophobic group (CH_3_) is used to replace the hydrophilic group (-OH) on the surface of the Fe/MCM-41 catalyst, which makes the catalyst surface hydrophobic. Hydrophobicity has the following main advantages: First, the water that is generated with the FTO reaction will be timely desorbed, inhibiting the WGSR and reducing CO_2_ selectivity. Secondly, exposure of more active sites for CO adsorption and dissociation increase the generation of active species and improve the CO conversion. Finally, the WGSR is limited, the H_2_/CO ratio will not increase, the hydrogenation capacity will decrease, and the selectivity of light olefins will increase, as shown in Figure 13 [105]. Javed et al. [13] prepared ZSM-5-supported iron-based microcapsule catalysts composed of an Fe/ZSM-5 core and silicalite-1 shell, as shown in Table 4. It was found that the silicate-1 layer was hydrophobic and non-acidic. The hydrophobicity causes the H_2_O to desorb from the surface of the catalyst, thereby inhibiting the WGSR and reducing the CO_2_ selectivity (Fe/ZSM-5: 20%, Fe/ZSM-5@S1-24(×2): 11%). The non-acidic shell of silicate-1 will reduce the acidity of the strong Brønsted acid in the core of the ZSM-5, inhibit the secondary hydrogenation of olefins, and improve the selectivity of light olefins (Fe/ZSM-5: 7% → Fe/ZSM-5@S1-24(×2): 11%). At the same time, the improvement of the selectivity of light olefins is also related to diffusion. This core–shell catalyst provides a customized closed reaction environment. The reactants reach the Fe/ZSM-5 nuclear catalyst through the silicate rock-1 shell, where they react to form intermediates or products. In order to leave the catalyst, all the intermediate nuclear and products must enter the surface channel of the silicate rock-1 shell. Due to the space limitation effect and shape selectivity, long-chain hydrocarbons will be cracked and converted into light olefins that can pass. However, the shell of the catalyst will also limit the activity of the catalyst to a certain extent. The decrease in CO conversion (Fe/ZSM-5: 53% → Fe/ZSM-5@S1-24(×2): 43%) is mainly due to the silicalite-1 layer covering the iron active sites dispersed on the ZSM-5 core.

In addition to the above different hydrophobic modified catalysts, there is another sense of functional modified catalysts. Recently, researchers have proposed a new coupling reaction to control the conversion of syngas [106]. The idea is to carry out CO activation dissociation and C–C coupling on the two active sites, and then integrate the two active sites into one, that is, a bifunctional catalyst. The catalyst design concept of the oxide–zeolite-based composite (OXZEO) enables direct syngas conversion to mixed light olefins with a selectivity reaching 80% and to ethylene with a selectivity of 83% among the hydrocarbons [107].

**Figure 14:**
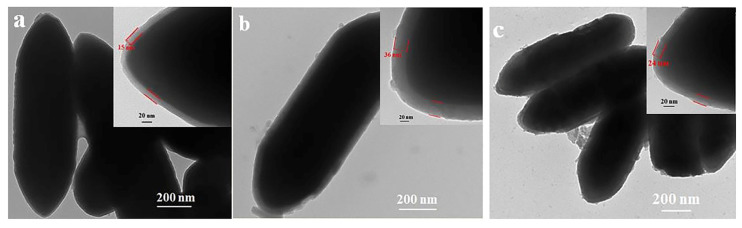
TEM images of the fresh catalysts for (**a**) Fe_2_O_3_@SiO_2_, (**b**) A-Fe_2_O_3_@SiO_2_-(CH_3_)_3_, (**c**) B-Fe_2_O_3_@SiO_2_-(CH_3_)_3_ [11].

The morphology of the synthesized catalyst particles is characterized using a transmission electron microscope and a scanning electron microscope, as shown in Figure 14 [11]. Figure 14 shows that the catalyst supported on SiO_2_ has an elliptical structure. There are still some structures such as microspheres [13]. Although the morphologies are different, the core surface of the Fe is covered with a silicon shell, which can be regarded as an important bridge and link between the Fe and -CH_3_, realizing the hydrophobic modification of the Fe-based catalysts. After the methyl modification, the shell thickness will increase but the morphology of the catalyst is similar, indicating that the stability is good and that the surface modification will not destroy the core–shell structure of the catalyst.

By adding hydrophobic groups -CH_3_ on the surface of the metal oxides and the molecular sieves, the interaction between the metal oxides and the active components, the surface properties of the support, and the acidity and alkalinity of the molecular sieve surface can be changed to improve the reaction performance of the catalyst. The addition of hydrophobic groups inhibits the interaction between the metal oxide and the active component, increases the reduction of the active component, provides more active sites, and increases CO conversion. However, the surface of the support will also form a large steric hindrance, reducing the number of active sites, thereby reducing CO conversion. Hydrophobic modification inhibits the acid strength of the acid sites in molecular sieves, reduces the secondary hydrogenation capacity of olefins, and increases the selectivity of light olefins.

### 6.3. Amphiphobic Modification

In the amphiphobic modification, hydrophobicity can inhibit the adsorption of H_2_ and WGSR, and reduce CO_2_ selectivity. Oleophobic modification can inhibit the secondary hydrogenation reaction of olefins and improve the selectivity of light olefins. Amphiphobic modification methods mainly include etching [108], sol–gel method [109], etc., but they are expensive and take a long time, which is not conducive to industrial research. As we all know, proper surface roughness and low surface energy are the keys to creating a hydrophobic and oleophobic surface [110], while coating fluorine atoms on the catalyst surface can have low surface energy, achieving the purpose of hydrophobic and oleophobic [14]. Guo et al. [111] coated fluoride on the surface of SiO_2_ nanoparticles to form a thin film. It was found that the contact angles of water and hexadecane were 161° and 156°, respectively, which indicates that the surface of silica sol is hydrophobic and oleophobic. Li et al. [112] also used the same method to attach a fluoride coating on the surface of aluminum alloy. It was found that the contact angles of water and hexadecane reached 162° and 152°, respectively, which is consistent with the above-mentioned literature structure and also has amphiphobic properties. Therefore, the addition of fluorine atoms is of great significance to the amphiphobic modification of the catalyst.

**Figure 15:**
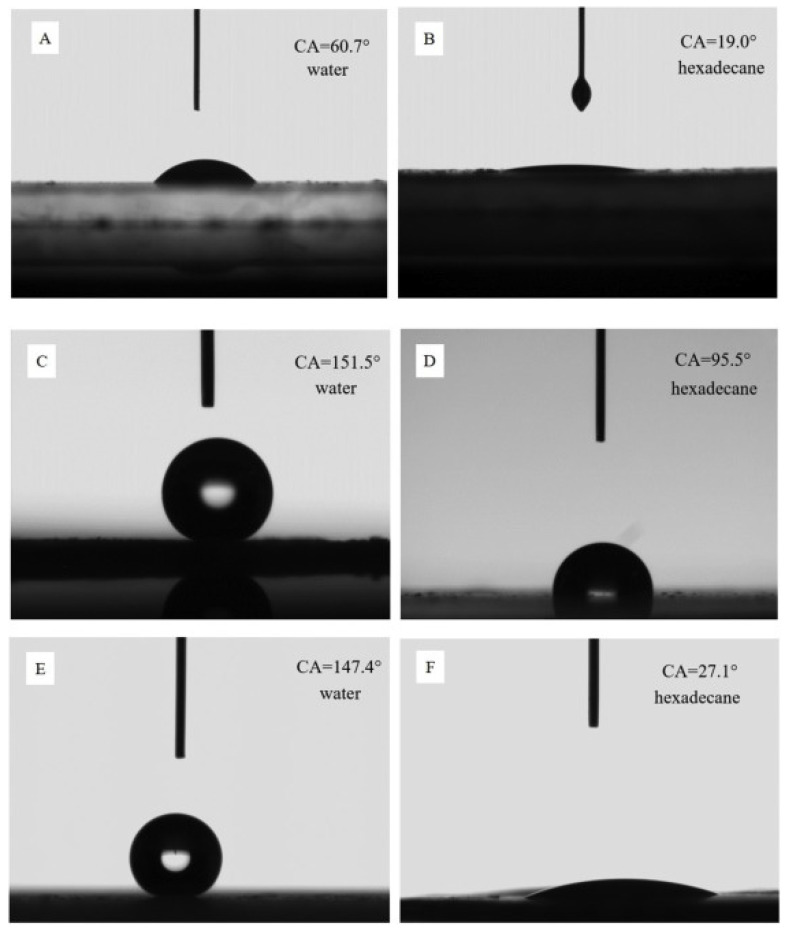
Water and hexadecane contact angles of fresh Fe_3_O_4_@SiO_2_ (**A**,**B**), Fe_3_O_4_@SiO_2_–PFTS (**C**,**D**), and spent Fe_3_O_4_@SiO_2_-PFTS (**E**,**F**) samples [113].

In the FTO reaction, the addition of fluorine atom also has hydrophobic and oleophobic surfaces. Yan et al. [113] prepared the core–shell Fe_3_O_4_@SiO_2_-PFTS catalyst with the amphiphobic modification, as shown in Table 4 (Fe_3_O_4_@SiO_2_ and Fe_3_O_4_@SiO_2_-PFTS). It was found that the contact angle of H_2_O in the modified Fe_3_O_4_@SiO_2_-PFTS catalyst changed from 60.7° hydrophilic to 151.5° hydrophobic, and the CA of hexadecane changed from 19.0° lipophilic to 95.5° oleophobic, as shown in Figure 15A–D. After the reaction, the CO_2_ selectivity decreased from the previous 44% to 12%, indicating that the WGSR is obviously inhibited. At the same time, the CO conversion is basically unchanged, the light olefin selectivity increased from 17% to 22%. It still had good stability after 120 h operation. The reason why CO is basically similar is that there is a strong interaction between Fe and SiO_2_ in the Fe_3_O_4_@SiO_2_ catalyst, which will inhibit the reduction and carbonization of the catalyst and reduce CO conversion. The Fe_3_O_4_@SiO_2_-PFTS catalyst inhibits CO adsorption and reduces CO conversion due to the steric hindrance formed by its core–shell structure. The reason for the increase in the selectivity of light olefins is that the modified steric hindrance and the WGSR will inhibit the secondary hydrogenation reaction of olefins and increase the selectivity of light olefins.

However, with the increase in reaction time, it was found that the surface of the Fe_3_O_4_@SiO_2_-PFTS catalyst changes from oleophobic to lipophilic. This is due to the increase in the steric hindrance of the catalyst, which will promote the reduction of the pore size so that the hydrocarbons are gathered on the surface of the catalyst and cannot be rapidly diffused. As a result, the increase in selectivity of light olefins caused by the increase in lipophilicity is low, as shown in Figure 15F. Therefore, it is necessary to increase the pore structure of the catalyst before the reaction, adjust the pore size of the catalyst, and ensure that the surface of the catalyst is still amphiphobic after the reaction.

**Table 4:** Summary of FTO performance of of Fe-based catalysts after hydrophobic and amphiphobic modification.

Catalysts	Temperature (K)	Pressure (MPa)	H_2_/CO	$X_{\mathrm{CO}}$ (%)	$S_{C_{2}{}^{=}{\sim C}_{4}{}^{=}}$ (%C mol)	$S_{{\mathrm{CO}}_{2}}$ (%)	Yield	Reference	Eqs.
Fe_2_O_3_@SiO_2_	593	1.5	2	96	8	40	8	[11]	(8) ^a,b^
A-Fe_2_O_3_@SiO_2_-(CH_3_)_3_	593	1.5	2	54	20	5	11	[11]	(8) ^a,b^
B-Fe_2_O_3_@SiO_2_-(CH_3_)_3_	593	1.5	2	50	18	5	9	[11]	(8) ^a,b^
FeMnO_x_	593	2.0	2	49	31	17	15	[12]	(4) ^b^
FeMnO_x_@SiO_2_-8.2-(CH_3_)_3_	593	2.0	2	51	55	5	28	[12]	(4) ^b^
FeMnO_x_@SiO_2_-13.1-(CH_3_)_3_	593	2.0	2	45	40	12	18	[12]	(4) ^b^
Fe/ZSM-5	533	1.0	2	53	7	20	4	[13]	(4) ^b^
Fe/ZSM-5@S1-24(×2)	533	1.0	2	43	11	11	5	[13]	(4) ^b^
Fe_3_O_4_@SiO_2_	593	1.5	2	34	17	44	6	[113]	(8) ^a,b^
Fe_3_O_4_@SiO_2_-PFTS	593	1.5	2	33	22	12	7	[113]	(8) ^a,b^

^a^: In the calculation of Equation (8), C_3_H_6_ and C_5_H_10_ are used to represent C_2_~C_4_ and C_5_^+^, ^b^: Results are not based on 1 g Fe.

## 7. Conclusions and Future Prospects

With an FTO reaction, the Fe-based catalyst has attracted much attention because of its low cost and large storage capacity. However, the product distribution follows the ASF distribution law, the product distribution range is wide, and the selectivity to light olefins is low. As the WGSR is high, it is easy to produce more CO_2_, which reduces the carbon utilization rate. Moreover, the large amount of H_2_O can easily cover the active sites of the catalyst and reduce the CO conversion of the catalyst, which is not conducive to large-scale industrial production. Therefore, hydrophobic modification can improve the adsorption and desorption of water in the catalyst byproduct, enhance the dispersibility and reducibility of the catalyst, and then improve the selectivity of CO_2_ and light olefins.

SiO_2_ supports can control the WGSR and the selectivity of light olefins to a certain extent because of their pore size, surface acidity, and alkalinity, but their effect is not significant. Hydrophobic modification can inhibit the WGSR and effectively reduce CO_2_ production, while oleophobic modification can improve the selectivity of light olefins in theory. However, they will reduce the stability of the catalyst, resulting in catalyst deactivation, reducing the CO conversion. In addition, the preparation conditions of amphiphobic modifications are complex and harsh, the mechanical properties of hydrophobic coatings are poor and easy to destroy, and the yield of light olefins and the stability of catalysts are not well controlled. Because of its unique acidic center and shape-selective performance, the molecular sieve can reduce the secondary hydrogenation reaction of olefins and improve the selectivity of light olefins. However, control of the acidic sites and the pore structure of a molecular sieve is random. Once the pores are small, the mass transfer of the reactants or products will be limited and will reduce the CO conversion and light olefin selectivity. Carbon supports can improve the selectivity of light olefins by adding promoters, changing surface acidity, alkalinity, and N doping. However, carbon supports are not resistant to high temperatures. If the temperature is too high, they can easily react with oxidizing substances such as oxygen. Their mechanical strength is not high, they wear faster, and their replacement cost is relatively high.

To sum up, we will conduct further research on the following aspects: (1) Poor hydrophobic stability. Hydrophobic modification and amphiphobic modification mainly combine hydrophobic substances on the micro-nano structure of the catalyst to make it hydrophobic. However, due to the poor adhesion between the hydrophobic material and the basement, friction and other interactions are prone to occur in the reaction, resulting in the shedding of hydrophobic materials and no hydrophobic properties. Therefore, it is necessary to increase the adhesion of the hydrophobic coating, strengthen the anti-aging performance of hydrophobic surface, and improve the stability, which is the main problem to be solved in the future. (2) The accurate construction of pore diameter. In the process of traditionally modified catalysts, the pore size is directly related to the selectivity of light olefins. A smaller pore size will form blockage and coke, resulting in the deactivation of the catalyst; when the pore size is large, C_5_^+^ will be removed directly without further cracking. Therefore, the accurate construction of the pore structure is of great significance to improve the selectivity of light olefins. (3) In most of the previous literature, there is no standard benchmark for the calculation and measurement of the catalyst reaction performance. The reaction performance of some catalysts is expressed by the hydrocarbon distribution, and some catalysts are expressed by hydrocarbon selectivity. The above two calculation methods are based on the quality of the catalyst or the quality of the active component iron. Therefore, the catalyst reaction performance can not be compared uniformly. In this paper, selectivity is used as the calculation method, and a relative comparison is made based on the mass of the active component iron. However, due to the lack of relevant values in some of the literature, some data cannot be calculated and converted in this paper. Therefore, unifying the calculation method and the measurement standard is suggested, so as to facilitate the comparison under a relatively unified benchmark in the subsequent literature calculation. (4) Further development. In recent years, due to global warming and environmental degradation, effectively reducing CO_2_ emissions and promoting ecological and environmental protection has become the focus. Hydrophobic modification can effectively reduce the byproduct CO_2_ in the process of the FTO reaction and solve the problem from the root. At present, there are few types of research on the modification of Fe-based catalysts and the hydrophobic modification of other metal supports. The hydrophobic modification of other metal oxides needs to be further studied.

## Data Availability

Data available in the publicly accessible repositories.

## Supplementary Materials

The following supporting information can be downloaded at: https://www.mdpi.com/article/10.3390/molecules28237749/s1, Table S1: Summary of FTO performance of iron catalysts supported on carbon material.
