# Supplementary material for: Research Progress on the Effects of Support and Support Modification on the FTO Reaction Performance of Fe-Based Catalysts
Source: Molecules. 2023 Nov 24;28(23):7749. doi: 10.3390/molecules28237749 (PMC10708045; doi:10.3390/molecules28237749)
Supplement: Supplementary file 1 [file molecules-28-07749-s001.zip › molecules-2712346-supplementary.pdf]

Table S1. Summary of FTO performance of iron catalysts supported on carbon material.

Catalysts	Temperature (K)	Pressure (Mpa)	H_2/CO	X_{CO} (%)	S_{C_n} (%C mol)	S_{CO_2} %)	Yield	Reference	Eqs.
Fe/AC	513	2.0	2.0	32	37	5	12	[77]	(7) [b]
Kn/Fe/AC	513	2.0	2.0	62	8	14	5	[77]	(7) [b]
（FeKn）/AC	513	2.0	2.0	86	6	19	5	[77]	(7) [b]
Fe-AC	593	2.0	1.0	62	7	42	4	[79]	(8) [a,b]
Fe-2MnK-AC	593	2.0	1.0	97	15	45	15	[79]	(8) [a,b]
Fe-AC	593	2.0	1.0	65	9	62	6	[81]	(8) [a,c]
FeN-10MnK-AC	593	2.0	1.0	39	19	47	7	[81]	(8) [a,c]
In-Fe/CNTs	543	2.0	2.0	85	---	39	---	[85]	---
Out-Fe/CNTs	543	2.0	2.0	79	---	40	---	[85]	---
Fe/CNTs	573	1.0	1.0	30	---	---	---	[86]	(7) [b]
Fe/CNTs-D	573	1.0	1.0	40	24	32	10	[86]	(7) [b]
FeMn5/CNTs-D	573	1.0	1.0	42	19	42	8	[86]	(7) [b]
Fe/MnK-CNTs	543	2.0	1.0	23	32	36	7	[87]	(7) [b]
Fe/CNTs	573	0.1	1.0	9	30	17	3	[88]	(7) [c]
Fe/NCNTs	573	0.1	1.0	14	38	19	5	[88]	(7) [c]
FeMgK1/rGO	613	2.0	1.0	59	22	41	13	[101]	(7) [c]
Fe/rGO	613	2.0	1.0	58	9	49	5	[102]	(7) [c]
FeK1/rGO	613	2.0	1.0	58	23	49	13	[102]	(7) [c]
FeK2/rGO	613	2.0	1.0	58	27	49	16	[102]	(7) [c]

[a]: In the calculation of Equation (8), C_3H_6 and C_5H_{10} are used to represent C_2~C_4 and C_5^+, [b]: Results are not based on 1g Fe, [c]: Results are based on 1 g Fe, ---: No original data.
